# A Case of Prolonged Catatonia Caused by Sjögren's Syndrome

**DOI:** 10.1155/2020/8881503

**Published:** 2020-11-03

**Authors:** Takahiko Inagaki, Kotaro Kudo, Naoki Kurimoto, Takashi Aoki, Kenichi Kuriyama

**Affiliations:** ^1^Biwako Hospital, 1-8-5, Sakamoto, Otsu, Shiga 520-0113, Japan; ^2^Department of Psychiatry, Shiga University of Medical Science, Seta Tsukinowa-cho, Otsu, Shiga 520-2192, Japan; ^3^Sanseikai Hospital, 1185, Uenohara, Uenohara, Yamanashi 409-0112, Japan; ^4^Shigasato Hospital, 1-18-41, Shigasato, Otsu, Shiga 520-0006, Japan; ^5^Shiga Hachiman Hospital, 744, Takakai-cho, Omihachiman, Shiga 523-0891, Japan; ^6^Department of Sleep-Wake Disorders, National Institute of Mental Health, National Center of Neurology and Psychiatry, 4-1-1, Ogawa-Higashi, Kodaira, Tokyo 187-8553, Japan

## Abstract

Sjögren's syndrome (SS) is a chronic autoimmune disorder, often associated with some neuropsychiatric symptoms as well as systemic lupus erythematosus. Although catatonia is frequently reported in patients with systemic lupus erythematosus, it has been rarely reported in patients with SS. Herein, we present a case of SS with catatonia effectively and safely treated with modified electroconvulsive therapy (ECT). A 58-year-old woman showed prolonged catatonia and depressive mood along with pathologically dried eye and mouth. Based on physical findings and blood tests, she was diagnosed with SS. Because of the presence of pressure sores, we were unable to perform lumbar puncture for the diagnosis of abacterial encephalitis. Alternatively, single-photon emission computed tomography of her brain revealed multifocal hypoperfused areas in the parietotemporal region. Consequently, we performed ECT for the treatment of catatonia comorbid with SS. Following 20 sessions of ECT, the catatonia was improved without obvious adverse effects. One week after the last ECT, elevated levels of interleukin-6 were identified in the cerebral fluid. After receiving steroid pulse therapy, she has not experienced catatonia for more than 5 years. SS can cause catatonia, and ECT is a safe and effective option for the treatment of catatonia with SS.

## 1. Introduction

Catatonia is a neuropsychiatric condition characterised by physical presentations ranging from profound immobility to excessive motor activity. It occurs as a clinical phenotype of various psychiatric, neurologic, or medical conditions especially affecting the central nervous system (CNS). It is often prolonged and accompanied by poor physical conditions; therefore, appropriate and prompt medical interventions are often required to avoid the development of pathological complications. Owing to its safety and effectiveness, electroconvulsive therapy (ECT) is a prime candidate for treating catatonia with some comorbid medical conditions [1].

It is well established that systemic lupus erythematosus (SLE) is associated with a risk of developing catatonia. SLE is often accompanied by some neuropsychiatric manifestations; thus, SLE comorbid with neuropsychiatric symptoms is termed neuropsychiatric SLE (NPSLE). Although catatonia is a rare neuropsychiatric symptom in SLE, it is also considered a symptom of NPSLE [2].

Sjögren's syndrome (SS) is also a chronic autoimmune disorder, which is often associated with psychiatric conditions (e.g., depression and anxiety disorders) [3]. Although SS shares a similar autoimmune pathogenesis with SLE and several cases of NPSLE with catatonia have been reported [2, 4, 5], there is limited knowledge regarding catatonia with SS. Herein, we report a case of prolonged catatonia comorbid with SS.

## 2. Case Presentation

A 58-year-old woman, without any past and family history of psychiatric illnesses, presented with symptoms of catatonia (e.g., extreme forward inclined posture, grim expression, extremely low volume of voice, and limited speech). She appeared to have acoustic hyperesthesia because loud conversations with individuals around her hurt her feelings. Hypopraxia resulted in some pressure sores on her hips. Subsequently, she developed additional depressive symptoms, including depressed mood, inhibition of thoughts, fatigue, low self-esteem, appetite loss, and sleep disturbances with marked psychosocial dysfunction. Her family informed the medical team that her catatonic symptoms were initially observed 7 months earlier and gradually worsened. She reported sharp pain in various parts of her body (mainly, the back), without any causes of the pain that directed her to consult us.

At the time of admission to our hospital, she had been receiving loxoprofen (prescribed by her family practitioner); however, she had discontinued treatment due to the absence of analgesic effects. Blood testing did not reveal signs of infection. Also, she did not show any signs of Parkinsonism other than the tonus enhancement of muscles and extreme forward inclined posture. Computed tomography and magnetic resonance imaging of her brain did not show any albocinereous or cerebrovascular abnormalities. These findings initially suggested that her catatonic symptoms may have been caused by major depressive disorder.

However, her family had not been aware of her depressive symptoms until the catatonic symptoms became obvious. In addition, we simultaneously noticed that her eyes and mouth were extremely dry. She did not present with any specific dermatological and mucosal symptoms of SLE. Pleuritis and pericarditis were excluded using ultrasonography of the heart. Urinalysis did not demonstrate proteinuria or cellular cast. She had leukopenia (1,500/mm^3^), but not anaemia, lymphopenia, or thrombocytopenia. Antiphospholipid antibodies, antibodies against native DNA, antinuclear antibodies, or antibodies against the Sm nuclear antigen were not detected. However, antibodies against Ro (SSA) were detected in her serum sample (128 U/mL). Schirmer's test showed positive hypolacrimation (2 mm in 5 min). Labial gland tissue biopsy showed several foci of lymphocytic sialoadenitis. According to the American College of Rheumatology Classification Criteria for SS [6] and Classification Criteria for SS of the American-European Consensus Group [7], we diagnosed this patient with SS.

**Figure 1:**
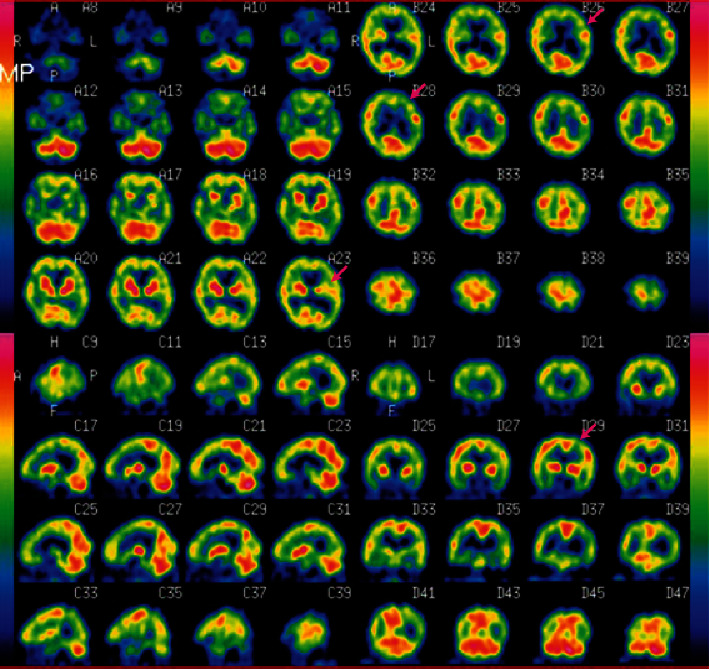
A head image of this patient obtained through single-photon emission computed tomography (SPECT). SPECT detected multifocal hypoperfused areas in the left parietotemporal region and left basal ganglia (red arrows).

Because of the presence of pressure sores, we were not able to perform lumbar puncture for the identification of inflammatory pathologies in the CNS. Single-photon emission computed tomography of the head showed multifocal hypoperfused areas (Figure 1). It has been reported that patchy diffuse hypoperfusion, as a manifestation of abacterial encephalitis, is commonly observed in patients with NPSLE [8]. Thus, we considered she had an autoimmune pathology in CNS and hypothesised that her catatonia was probably caused by SS rather than major depressive disorder. Subsequently, we performed modified electroconvulsive therapy (ECT) to treat her catatonia.

She received a 20-session course of ECT within a period of 10 weeks. There were no adverse effects that interfered with the treatment, except for minor temporal delirium. Her catatonia was obviously improved along with the other depressive symptoms, including appetite loss and decline in locomotor activity. One week after the final ECT session, her pressure sores had completely resolved, and we were able to collect cerebrospinal fluid through a lumbar puncture for cytological examination. The results showed an increased cell count (10/*μ*L), elevated IgG levels (14.3 mg/dL), and elevated interleukin-6 levels (5.14 pg/mL). The specific gravity and protein levels were within the normal limits. Based on these observations, we confirmed the diagnosis of neuropsychiatric SS.

Following steroid pulse therapy, she has not experienced catatonia for more than 5 years without the need for psychiatric treatments (e.g., antidepressants and ECT).

We obtained written informed consent for publication from the patient.

## 3. Discussion

We noted two important clinical observations in this case. Firstly, SS can be occasionally accompanied by catatonia. Moreover, delayed detection and inadequate treatment of catatonia in the early phase could prolong and deteriorate the general condition of the patient. In this case, we treat the prolonged catatonia along with depressive symptoms using steroid pulse therapy and its maintenance therapy after a course of ECT. The patient has been free of catatonia for a period of >5 years. The therapeutic course suggested that catatonia with depressive episodes was a consequence of SS pathology. Although psychiatric symptoms have been commonly reported in SS [3], to the best of our knowledge, only one case of catatonia comorbid with SS has been previously reported [9].

Secondly, ECT can be safe and effective option for the treatment of prolonged catatonia caused by SS. SS shares a similar autoimmune pathogenesis with SLE; hence, catatonia with SS could be treated with ECT via similar improvement mechanisms to those observed for SLE [2, 4, 5]. Malur et al. suggested that ECT is effective for the treatment of prolonged catatonia with complex medical comorbidities, but may require a higher number of treatment sessions compared with therapy for simple catatonia [10]. This case also required more ECT sessions to achieve remission of catatonia than those needed for the treatment of major depression. However, the course of ECT did not lead to any major adverse events.

This case implied that various chronic autoimmune disorders potentially involve CNS pathology associated with neuropsychiatric symptoms, including catatonia. While diagnosing autoimmune disorders with psychiatric symptoms, highly invasive tests, such as cerebrospinal fluid examination, are required. Thus, when the patient is debilitated, as in cases with catatonia, physicians must sometimes prioritise empirical therapy over definitive diagnosis to save the patient's life. The empirical administration of ECT may be relatively safe and effective for the treatment of catatonia comorbid with chronic autoimmune disorders. Moreover, it may be helpful in promoting the diagnostic process of comorbid autoimmune disorders responsible for catatonia.

## 4. Final Diagnosis

The final diagnosis was catatonia associated with Sjögren's syndrome.

## Data Availability

The clinical data used to support the findings of this study are included within the article.
